# Spastic Paraplegia Type 78 Associated With *ATP13A2* Gene Variants in Compound Heterozygosity

**DOI:** 10.1002/mgg3.70073

**Published:** 2025-02-11

**Authors:** R. Bermejo Ramírez, N. Villena Gascó, L. Ruiz Palmero, G. A. Ribes Bueno, E. Seiti Yamanaka, J. D. Arroyo Andújar

**Affiliations:** ^1^ Progenie Molecular S.L.U. Valencia Spain

**Keywords:** *ATP13A2*, hereditary spastic paraplegia, pathogenic variant, spastic paraplegia type 78, whole‐exome sequencing

## Abstract

**Background:**

Spastic Paraplegia Type 78 (SPG78) is a rare form of hereditary spastic paraplegia (HSP), mainly characterized by late‐onset lower‐limb spasticity, muscle weakness, and in some cases cerebellar dysfunction and cognitive impairment. Understanding its genetic background is essential to distinguish it from other autosomal recessive types of HSP.

**Methods:**

A pathogenic variant screening in a Spanish HSP patient was carried out by whole‐exome sequencing, followed by a software filtering process and validation of candidate variants by Sanger sequencing. The pathogenicity of the selected variants was evaluated by In Silico predictions and a segregation analysis including the proband and 16 family members.

**Results:**

Two variants in the *ATP13A2* gene, predicted to have damaging effects by In Silico analyses, were considered potentially pathogenic: NM022089.4:c.649G>A (rs199961048), previously associated with SPG78 but with uncertain clinical significance, and NM_022089.4:c.2097delC, an unreported variant. The segregation analysis revealed that both variants were present in compound heterozygosity in the proband and two affected siblings, while unaffected relatives carried only one or none of the variants.

**Conclusion:**

These findings suggest that the two variants are pathogenic when occurring in compound heterozygosity and, therefore, should be included in the genetic spectrum of SPG78 diagnosis.

## Graphical abstract

In this work, a pathogenic variant screening was performed in a hereditary spastic paraplegia patient by whole‐exome sequencing. Two potentially pathogenic ATP13A2 variants were selected and submitted to a segregation analysis with 16 family members, indicating that these variants are pathogenic for spastic paraplegia type 78 when occurring in compound heterozygosity.

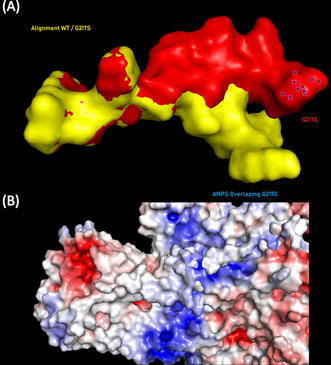

## 1 Introduction

Hereditary spastic paraplegia (HSP) is a genetically and clinically heterogeneous group of upper motor neuron diseases, characterized by lower extremity weakness and progressive spasticity due to the motor‐sensory axon degeneration, that predominantly affects the distal ends of the long central nervous system (McDermott et al. 2000; Giudice et al. 2014). More than 80 known types of HSP have been reported to this date (Hedera 2000), with variable symptom onset, severity, and inheritance mechanisms (Meyyazhagan and Orlacchio 2022).

Due to the variability on symptoms, diagnosing HSP based solely on the clinical manifestations can be a challenging task. Genetic testing has shown to be critical in order to confirm the specific diagnosis, through the association of the clinical features with pathogenic variants in HSP‐related genes. Furthermore, it allows the physician to perform genetic counselling and inform the possibility of new HSP cases in a family.

The object of this study was to identify pathogenic variants in a proband affected by HSP. It was achieved by performing whole‐exome sequencing, selecting candidate variants by software analysis and inferring their pathogenicity by a segregation analysis with family members.

## 2 Materials and Methods

### 2.1 Ethical Compliance

This study was designed and conducted in accordance with the ethical standards of the 1964 Helsinki Declaration and Spanish Law 14/2007 on Biomedical Research. It was approved by the Research Ethics Committee of the Universitat de València (10 September 2020). A written informed consent to perform genetic tests and publish the results was signed by all participants of this study. In the case of minors or incapacitated subjects, the consent was signed by the legal guardian.

### 2.2 Pathogenic Variant Screening and Segregation Analysis

Blood samples from the proband were collected in EDTA tubes for performing whole‐exome sequencing, whereas oral swab samples were collected from the family members for Sanger sequencing. DNA purification was performed with a MagCore Super extractor instrument (RBC Bioscience's Corp, Taiwan). A MagCore Viral Nucleic Acid extraction kit (Low PCR inhibition MVN400‐04) was used for the blood samples and a Genomic DNA Tissue Kit (MagCore DNA Tissue Kit MGT‐02) for the oral swabs.

The whole‐exome sequencing of the proband's DNA was performed using the SureSelect Human All Exon V6 capture kit (Agilent Technologies, United States) and HiSeq 4000 platform (Illumina, United States). In order to discard nonpathogenic variants, a process of data filtering, analysis and interpretation was carried out using the BioVisor NGS software (Progenie Molecular, Spain) and its own pipeline method. After this filtering phase, the resulting variants were interpreted individually, based on available literature data, clinical features and expression effect by bioinformatic predictions. The details of the variant detection and selection can be found in the Supporting Information.

The single nucleotide variants of interest were validated by Sanger sequencing with a BigDye Terminator kit (Applied Biosystems, United States) and subsequently resolved with the ABI PRISM 310 Genetic Analyzer (Applied Biosystems) and SeqStudio Genetic Analyzer (Applied Biosystems). This validation was carried out for the proband and 16 family relatives in a segregation analysis, in order to evaluate the inheritance mechanism and pathogenicity of the suspected variants.

### 2.3 Bioinformatics Analysis

The damaging effect prediction of the candidate variants was carried out using the Sorting Intolerant From Tolerant (SIFT) (Ng and Henikoff 2001; Hu and Ng 2013), Polymorphism Phenotyping version 2 (PolyPhen‐2) (Adzhubei et al. 2013), Combined Annotation‐Dependent Depletion (CADD v.1.7) (Schubach et al. 2024), Rare Exome Variant Ensemble Learner (REVEL) (Ioannidis et al. 2016) and Alpha Missense (Cheng et al. 2023) tools. The AlphaFold 2 (Jumper et al. 2021; Varadi et al. 2024) was employed to create the 3D model of the wild‐type and variant‐associated ATP13A2 structures.

## 3 Results

### 3.1 Case Description

A clinical history analysis revealed that the proband started exhibiting gait disturbance and occasional falls at the age of 52. The symptoms worsened progressively, with the manifestation of spasticity in the lower limbs, lateral Babinski sign, Dupuytren's contracture, and severe generalized action myoclonus verified at Age 61. A wheelchair was necessary to assist locomotion since Age 62 and at 64 autonomous ambulation became impossible. At the age of 65, the patient experienced episodes of generalized epileptic seizures, which were successfully suppressed with lamotrigine therapy. He died at 69 years old in consequence of a multiple organ failure.

At the time of presentation, the patient was 68 years old. He showed mixed pyramidal and extrapyramidal hypertonia in the upper limbs, presenting a dystonic posture of flexion in both fists, only partially reversible, along with a tendency toward elbow flexion. Spastic hypertonia was exhibited in the lower limbs, with bilateral extensor plantar reflexes. No contractures were observed in the upper or lower limbs. A slight dysmetria was noted. He presented dysphagia, hypophonia and severe dysarthria, with reduction in spontaneous verbal fluency. There was a disturbance in the frontal saccadic eye pursuit, showing slowed and hypometric movements, with preservation of parietal‐initiated saccadic eye motion and head movement. The patient did not exhibit nystagmus, nor facial dystonia or tongue atrophy, with normal tongue protrusion. The spasticity and hypertonia were attenuated by treatment with levetiracetam and clonazepam. A mild‐to‐moderate cognitive impairment was exhibited, although a reliable evaluation was hindered by the patient's communication limitations and slowness.

A brain magnetic resonance revealed cortical–subcortical and cerebellar atrophy. In the T1 sequence, there was a mild hyperintensity of the globus pallidus. In the gradient‐echo sequence, a moderate paramagnetic deposition was observed in the globus pallidus and the substantia nigra, although not clearly pathological. Computed tomography scans revealed no calcifications and a DaTscan showed normal results.

The proband's family consisted of 16 members from the autonomous community of Catalonia (Spain). His parents were asymptomatic and no consanguinity was reported in the family. The patient had four siblings, two unaffected and two others diagnosed with HSP, one of them also presenting myoclonic tremor. Both affected siblings started showing symptoms at the fifth decade of life and died at ages 69 (subject II‐4) and 65 (subject II‐7) due to COVID‐19 pneumonia. All three affected siblings presented a late onset and fast disease progression. The other family members (Subjects III‐1 to III‐12 and IV‐1 to IV‐3) did not suffer from HSP symptoms.

### 3.2 Variant Analysis

**FIGURE 1:**
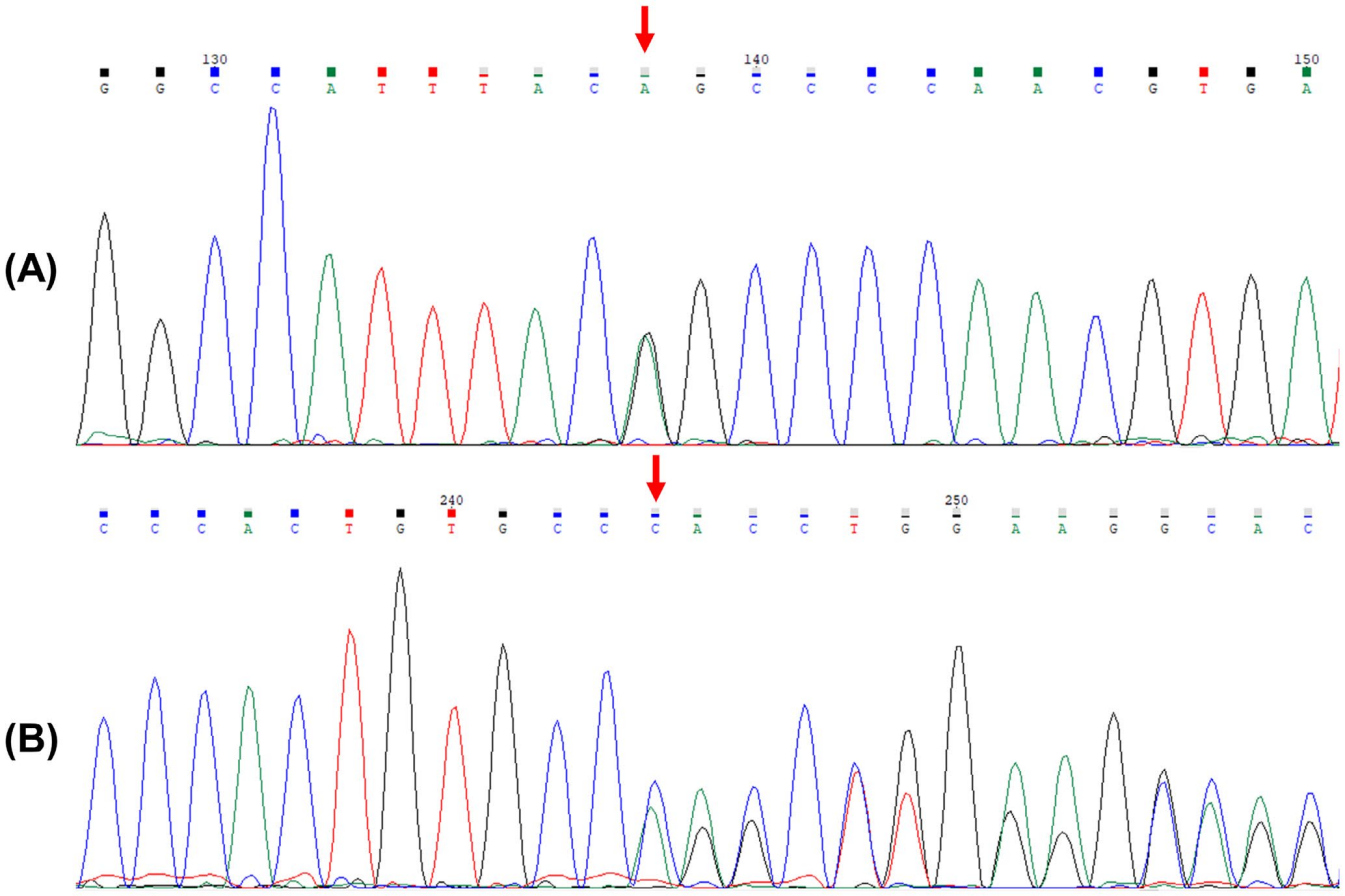
Sanger sequencing electropherogram of two variants in the *ATP13A2* gene (NM_022089.4): (A) c.649G>A (p.Gly217Ser); (B) c.2097delC (p.Pro699fs). Due to the frameshift, the nucleotide‐call after this variant represents the overlapped sequences of wild‐type and mutant alleles. A red arrow indicates the mutated nucleotide.

The whole‐exome sequencing analysis and data filtering by the BioVisor NGS software resulted in 19 variants that were individually interpreted. Two of them, located in the *ATP13A2* gene (OMIM 610513), were considered potentially pathogenic variants: NM_022089.4:c.649G>A (NP_071372.1:p.Gly217Ser, rs199961048) and NM_022089.4:c.2097delC (NP_071372.1:p.Pro699fs), identified in compound heterozygosity (Figure 1).

**FIGURE 2:**
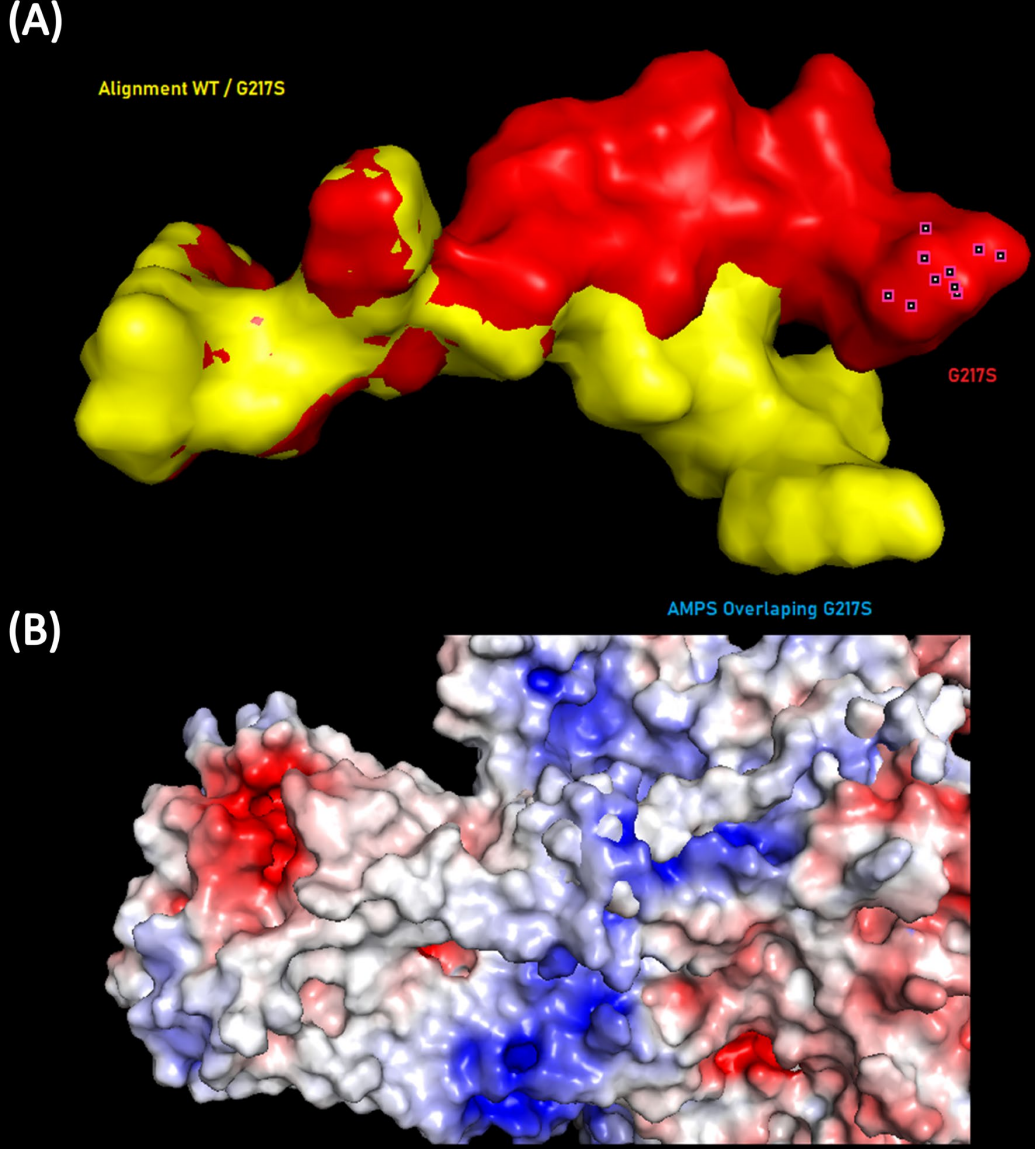
(A) Three‐dimensional structure alignment between the wild‐type structure of the ATP13A2 protein (NP_071372.1) and the G217S variant. (B) Structural surface energy representation by the Alignment of Multiple Protein Sequences (AMPS).

The c.649G>A (p.Gly217Ser, rs199961048) variant is located in exon 8 and was previously reported in the dbSNP database as being associated with Kufor‐Rakeb syndrome and SPG78 with uncertain significance. The frequency of this missense variant is very low in both the Exome Aggregation Consortium (ExAC, 0.002%) and the Genome Aggregation Database (gnomAD, 0.0013%). The In Silico predictions scores for rs199961048 were 0.01 by SIFT (deleterious), 0,978 by PolyPhen‐2 (probably damaging), 0.883 by REVEL (likely disease causing), 30 by CADD (likely pathogenic) and 0.393 by Alpha Missense (ambiguous). A comparison between the 3D structure of wild‐type and p.Gly217Ser variant (Figure 2) showed a light surface energy loss, which causes a slight torsion and protrusion of the 12 adjacent codons.

Regarding c.2097delC, located in exon 19, to this date this variant was unreported and no information about its clinical significance has been published. The variant is absent in the ExAC and gnomAD frequency databases. The deletion of a cytosine at nucleotide 2097 causes a frameshift that produces a premature stop codon, which had a CADD score of 25.5 (likely pathogenic). An In Silico analysis with indel SIFT tool predicted this variant to be damaging with a 0.858 confidence score and to cause nonsense‐mediated mRNA decay, leading to a null effect of the variant allele.

**FIGURE 3:**
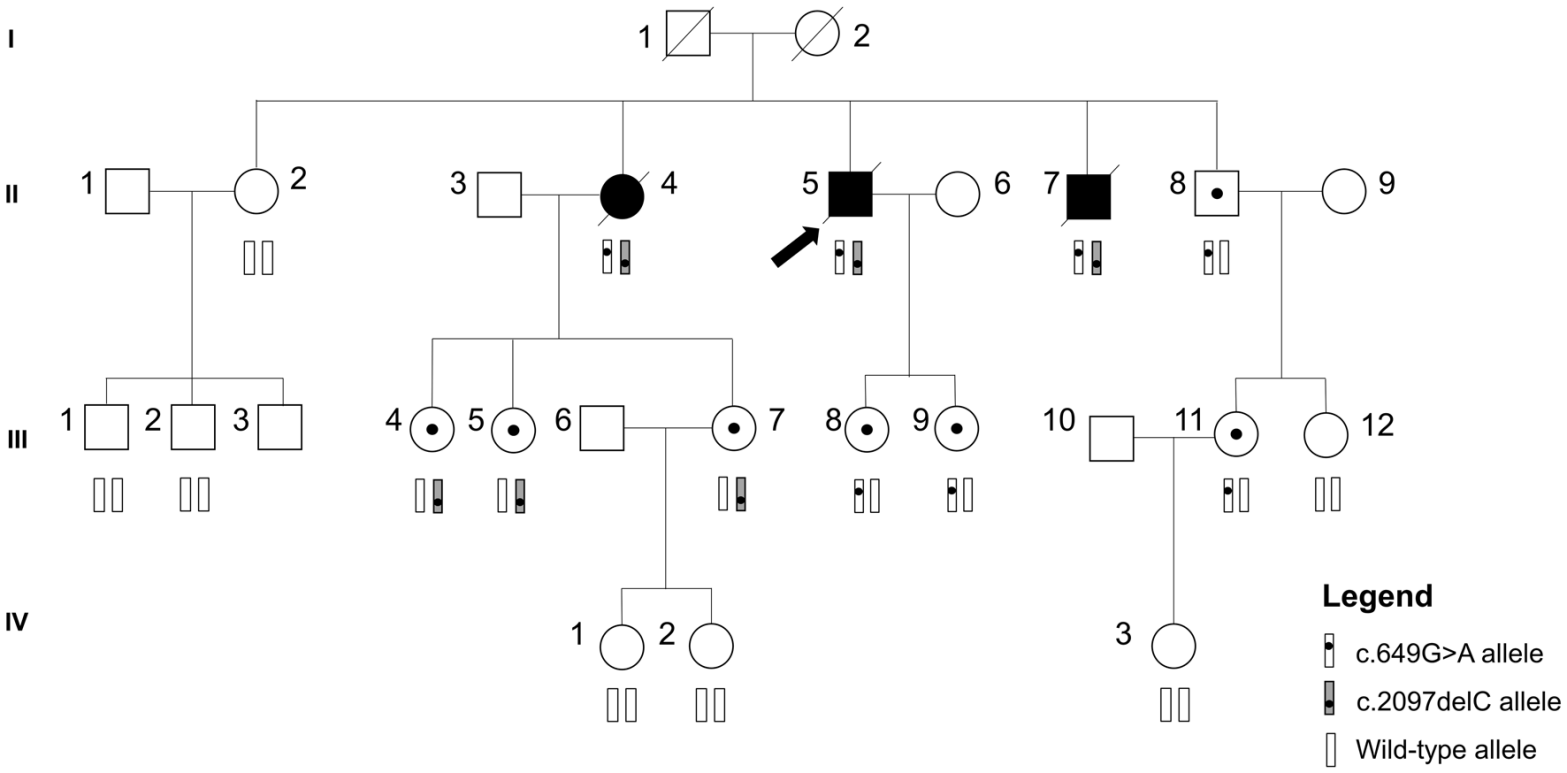
Family pedigree. Subject II.5 is the index case (arrow marked). Subjects II‐4, II‐5, and II‐7 were affected and all carried both *ATP13A2* variants (NM_022089.4: C.649G>A and c.2097delC). The proband's unaffected brother (Subject II‐8) only carried c.649G>A and his unaffected sister II‐2 did not carry any of the studied variants. The other tested relatives (daughters, nephews, and nieces of the index case and their offspring), all unaffected, carried only one or none of the variants. Square: Male; circle: Female; white marked: Unaffected; black‐marked: Affected; dotted‐marked: Unaffected carrier; slash‐marked: Deceased.

Sixteen relatives were analyzed by Sanger sequencing in order to evaluate the segregation of the HSP phenotype and the presence of the candidate variants. Figure 3 shows the family pedigree that summarizes the results for both suspected variants. All three affected subjects presented both variants in compound heterozygosity, suffering from similar clinical features, with onset after the fifth decade of life, affecting males and females. They showed global spasticity and hypertonia, with cerebellar signs, which fit with the phenotypes associated with variants in *ATP13A2* and spastic paraplegia 78. The individuals carrying only one or none of the variants did not show any symptoms of HSP. None of the studied variants was detected in the fourth generation. These results corroborate the pathogenic effect of the studied variants when they are both present in the same individual, revealing the autosomal recessive heritage of HSP in this family.

A third variant located in the *GDAP1* gene (NM_018972.2:c.487C>T) was detected in the proband in heterozygosity. As pathogenic variants occurring in this gene could cause autosomal recessive or dominant Charcot–Marie–Tooth disease, an additional Sanger sequencing analysis was performed for the two affected siblings in order to verify the variant's contribution to the disease. The variant was found in heterozygosity in Subject II‐4 and was absent in Subject II‐7, suggesting that it has a recessive mode of inheritance and does not segregate with the patients' phenotype.

## 4 Discussion

SPG78 is a rare form of HSP and only a few cases have been reported to this date. Since this family had a large number of members, it allowed to establish a relation between the *ATP13A2* variants and the manifestation of the HSP phenotype. Variants in *ATP13A2* were firstly associated in 2006 to Kufor‐Rakeb syndrome (OMIM 606693), also known as Parkinson disease 9 with dementia (PARK9), (Ramirez et al. 2006) and were later identified in neuronal ceroid lipofuscinosis patients (Bras et al. 2012). Nevertheless, some groups reported cases where *ATP13A2* variants caused a late‐onset, autosomal recessive type of HSP called spastic paraplegia Type 78 (SPG78; OMIM 617225) (Kara et al. 2016; van De Warrenburg et al. 2016; Estrada‐Cuzcano et al. 2017).

Regarding the effect of the studied variants, the damage caused by the c.2097delC variant is very prominent, as the protein is not expected to be expressed. However, this variant alone seems to be insufficient to cause SPG78, since the proband's parents were asymptomatic and the Subjects III.4, III.5, and III.7, who are c.2097delC heterozygous carriers, did not manifest the SPG78 clinical features. Other similar variants that generate a premature stop codon (c.3057delC and c.2473_2474insAAdelC) detected in the *ATP13A2* gene have been reported as pathogenic for PARK9 and SPG78 (Inzelberg et al. 2018; Estiar et al. 2020).

The damaging effect of c.649G>A is less clear, although the In Silico analyses predicted this variant to be likely pathogenic. The p.Gly217Ser change occurs in a topological cytoplasmic domain which seems to be unrelated to autophosphorylation or polyamine binding (Holemans et al. 2015; Tillinghast et al. 2021). However, as polyamines are the only verified substrate to this protein and the transport mechanism of other unknown substrates was not yet discovered, it is still difficult to determine how the conformational change generated by the p.Gly217Ser could influence the protein activity and the consequent physiological effects. Recently, this variant has been reported in a heterozygous patient as putatively contributing to a form of late‐onset Parkinson disease (Tejera‐Parrado et al. 2020).

The pathogenic effect of biallelic variants is not uncommon in the *ATP13A2* gene. Some groups found variants in compound heterozygosity related to SPG78 (c.1330C>T and c.3403C>T) (Estrada‐Cuzcano et al. 2017), PARK9 (IVS13+5G>A and c.3059delC, c.3176T>G and c.3253delC, c.3057delC, and c.1306+5G>A) (Brüggemann et al. 2010; Park et al. 2015) and in two subjects with mixed symptoms of SPG78 and PARK9 (c.2529+1G>A and c.3057delC) (Miranda et al. 2020). A close relation between these two conditions was observed and there is the possibility that they could be different manifestations of the same disorder. Further studies exploring the pathogenic mechanism of *ATP13A2* dysfunction could contribute to elucidate this connection.

## 5 Conclusion

This study suggests that both c.649G>A and c.2097delC variants identified in the *ATP13A2* gene in compound heterozygosity were responsible for the HSP in this family and, therefore, should be considered as pathogenic variants when present in the same individual. Thus, it is concluded that the identification of the two described *ATP13A2* variants should be included in routine analysis of HSP patients with similar symptoms, in order to improve their diagnosis. Nevertheless, although it is very likely that these variants are disease‐causing, the mechanism is not elucidated and functional protein studies are encouraged.

## Author Contributions

**R. Bermejo Ramírez:** investigation, writing – original draft, writing – review and editing. **N. Villena Gascó:** investigation. **L. Ruiz Palmero:** supervision, validation, writing – review and editing. **G. A. Ribes Bueno:** software. **E. Seiti Yamanaka:** visualization, writing – review and editing. **J. D. Arroyo Andújar:** conceptualization, methodology, validation, writing – review and editing.

## Conflicts of Interest

R. Bermejo Ramírez, N. Villena Gascó, L. Ruiz Palmero, G. A. Ribes Bueno, E. Seiti Yamanaka and J. D. Arroyo Andújar had a work contract with Progenie Molecular S.L.U. while executing this research.

## Supporting information


**Data S1.**.

## Data Availability

The data that supports the findings of this study are available from the corresponding author upon reasonable request. Raw data from the whole‐exome sequencing analysis is not available due to privacy or ethical restrictions. The NM022089.4:c.649G>A and NM_022089.4:c.2097delC variants were submitted to the ClinVar database under accession numbers SCV004171054.1 and SCV004171055.2, respectively.
