# Supplementary material for: Spastic Paraplegia Type 78 Associated With ATP13A2 Gene Variants in Compound Heterozygosity
Source: Mol Genet Genomic Med. 2025 Feb 11;13(2):e70073. doi: 10.1002/mgg3.70073 (PMC11814479; doi:10.1002/mgg3.70073)
Supplement: Supplementary file 1 — Data S1.. [file MGG3-13-e70073-s001.pdf]

Spastic paraplegia type 78 associated to *ATP13A2* gene variants in compound heterozygosity

R. Bermejo Ramírez,[1] N. Villena Gascó,[1] L. Ruiz Palmero,[1] G.A. Ribes Bueno,[1] E. S. Yamanaka,[1] J. D. Arroyo Andújar [1,*]

[1] Progenie Molecular S.L.U. Valencia, Spain.

*Corresponding author at: Progenie Molecular S.L.U. Edificio Progenie. Calle Valle de la Ballestera 56, 46015 Valencia, Spain. E-mail address: darroyo@progenie-molecular.com (J.D. Arroyo Andújar)

Supporting information

Whole-exome sequencing and variant selection

A whole-exome analysis was performed from an EDTA-blood sample from the proband. Whole-exome sequencing was carried out using a SureSelect V6-Post (Agilent, USA) capture kit and HiSeq 4000 platform (Illumina, USA). A total of 51,651,565 reads were obtained with a mean depth of 51,7X. Table S1 summarizes the variants detected in the proband's sample.

Table S1. Variants detected in a hereditary spastic paraplegia proband by whole-exome sequencing

Variant type	Detected number
Single nucleotide polymorphisms	91,389
Synonymous variants	11,780
Missense variants	11,209
Stop gained	102
Stop lost	38
Insertions/deletions	9,885
Frameshift variants	99
In-frame insertions	153
In-frame deletions	152

The BioVisor NGS software (Progenie Molecular, Spain) was employed to analyse the identified variants, by performing a filtering process based on 5,863 variants located in 901 genes related to neuromuscular diseases (Table S2). The software excluded benign variants by the following criteria: frequency ≥ 0.01; classified as benign in the ClinVar database; intronic variants, deletions, insertions and duplications at ± 2 nt; 5' UTR and 3' UTR variants at ± 2 nt; variants in non-coding exons and synonymous variants. 19 potentially pathogenic variants within 18 genes, all found in heterozygosity, were selected for individual analysis according to clinical features and pathogenic potential (Table S3).

Table S2. Genes included in the panel for neuromuscular diseases

A2M	ATP2B3	COL6A2	EIF2B5	GJB3	KCNJ18	MTFMT	PEX11B	PRX	SLC19A3	TDP2	WASHC5
AAAS	ATP2B4	COL6A3	EIF4G1	GLA	KCNJ2	MTM1	PEX12	PSAP	SLC1A3	TECPR2	WDR45
AARS	ATP7A	COLQ	ELOVL4	GLB1	KCNJ5	MTMR14	PEX13	PSEN1	SLC1A4	TENM4	WDR48
AARS2	ATP7B	COQ2	ELOVL5	GLDN	KCNMA1	MTMR2	PEX14	PSEN2	SLC20A2	TFG	WDR73
ABCA1	ATP8A2	COQ8A	ELP1	GLE1	KCNQ2	MTPAP	PEX16	PTRH2	SLC22A5	TGFB3	WDR81
ABCA7	ATXN1	COQ9	EMD	GLRA1	KCNQ3	MTTP	PEX19	PTS	SLC25A1	TGM6	WFS1
ABCB7	ATXN10	COX10	ENO3	GLRB	KCNT1	MUSK	PEX2	PUS1	SLC25A12	TH	WNK1
ABHD12	ATXN3	COX15	ENTPD1	GLUD2	KCTD17	MYBPC1	PEX26	PYCR2	SLC25A15	THAP1	WWOX
ABHD5	ATXN7	COX6A1	EPHA4	GM2A	KCTD7	MYF6	PEX3	PYGM	SLC25A19	TIA1	XK
ACAD9	AUH	CP	EPM2A	GMPPB	KDM5C	MYH14	PEX5	PYROXD1	SLC25A20	TK2	XPA
ACADL	B3GALNT2	CPLX1	EPRS	GNAL	KIAA0556	MYH2	PEX7	QDPR	SLC25A3	TMEM106B	XPC
ACADM	B4GALNT1	CPOX	ERBB3	GNAO1	KIAA0586	MYH3	PFKM	RAB39B	SLC25A4	TMEM126B	XPR1
ACADS	B4GALT1	CPT1C	ERBB4	GNB4	KIAA0753	MYH7	PFN1	RAB3GAP2	SLC25A46	TMEM138	YARS
ACADVL	B4GAT1	CPT2	ERCC2	GNE	KIDINS220	MYH8	PGAM2	RAB7A	SLC2A1	TMEM216	YARS2
ACER3	B9D1	CRAT	ERCC3	GOLGA2	KIF14	MYO18B	PGAP1	RAI1	SLC30A10	TMEM231	ZBTB42
ACOX1	BAG3	CRYAB	ERCC4	GOSR2	KIF1A	MYO9A	PGK1	RAPSN	SLC33A1	TMEM237	ZC4H2
ACTA1	BCAP31	CSF1R	ERCC5	GRID2	KIF1B	MYOD1	PGM1	RARS	SLC35A3	TMEM240	ZFR
ACVR1	BCS1L	CSPP1	ERCC6	GRM1	KIF1C	MYOT	PHGDH	RARS2	SLC39A14	TMEM43	ZFYVE26
ADAM10	BEAN1	CSTB	ERCC8	GRN	KIF21A	MYPN	PHKA1	RBCK1	SLC52A2	TMEM5	ZFYVE27
ADAR	BICD2	CTC1	ERGIC1	GSN	KIF5A	NAGLU	PHKA2	RBM7	SLC52A3	TMEM67	ZNF423
ADCY5	BIN1	CTDP1	ERLIN1	GYG1	KIF7	NAIP	PHKB	REEP1	SLC5A7	TNNI2	ZNF592
ADCY6	BSCL2	CTNND2	ERLIN2	GYS1	KLC2	NALCN	PHKG2	REEP2	SLC6A3	TNNT1	
ADGRG6	BVES	CTSA	ETFA	HACD1	KLHL24	NDRG1	PHYH	REPS1	SLC6A5	TNNT3	
ADH1C	C19orf12	CTSD	ETFB	HACE1	KLHL40	NDUFA1	PIBF1	RETREG1	SLC7A2	TNPO3	
ADRA2B	C21orf2	CWF19L1	ETFDH	HADH	KLHL41	NDUFA10	PIEZO2	RIPK4	SLC9A6	TOR1A	
ADSSL1	C5orf42	CYP27A1	ETHE1	HADHA	KLHL7	NDUFA12	PIGA	RNASEH2A	SLURP1	TOR1AIP1	
AFG3L2	C9orf72	CYP2U1	EXOSC3	HADHB	KLHL9	NDUFA2	PIGN	RNASEH2B	SMAD3	TPK1	
AGK	CA2	CYP7B1	EXOSC8	HARS	KMT2B	NDUFA4	PIGT	RNASEH2C	SMCHD1	TPM2	
AGL	CA8	DAG1	FA2H	HEXA	KY	NDUFA9	PIK3R5	RNASET2	SMN1	TPM3	
AGRN	CACNA1A	DAO	FAM126A	HEXB	L1CAM	NDUFAF5	PINK1	RNF168	SMPD1	TPP1	
AHI1	CACNA1B	DARS	FARS2	HIBCH	L2HGDH	NDUFAF6	PIP5K1C	RNF170	SNAP25	TRAPPC11	
AIFM1	CACNA1G	DARS2	FBLN5	HIKESHI	LAMA1	NDUFS1	PLA2G6	RNF216	SNCA	TREM2	
AIMP1	CACNA1S	DCAF17	FBN1	HINT1	LAMA2	NDUFS2	PLAA	RPGRIP1L	SNCB	TREX1	
AIMP2	CACNB4	DCAF8	FBN2	HK1	LAMB2	NDUFS3	PLAU	RPIA	SNTB1	TRIM2	
ALAD	CAMTA1	DCTN1	FBN3	HMBS	LAMP2	NDUFS4	PLEC	RRM2B	SNX14	TRIM32	
ALDH18A1	CAPN1	DCTN2	FBXL4	HNRNPA1	LARGE1	NDUFS7	PLEKHG2	RTN2	SOD1	TRIM54	
ALDH3A2	CAPN3	DDB2	FBXO38	HNRNPA2B1	LAS1L	NDUFS8	PLEKHG4	RUBCN	SORL1	TRIM63	
ALDOA	CASK	DDC	FBXO7	HOXD10	LDB3	NDUFV1	PLEKHG5	RXYLT1	SOX10	TRIP4	
ALG14	CASQ1	DDHD1	FDX1L	HPCA	LGI4	NEB	PLOD2	RYR1	SPART	TRMU	
ALG2	CAV3	DDHD2	FECH	HPRT1	LIMS2	NEFL	PLP1	SACS	SPAST	TRPA1	
ALG3	CAVIN1	DES	FGD4	HRAS	LIPT1	NEK1	PMM2	SAMD9L	SPEG	TRPC3	
ALS2	CC2D2A	DGAT2	FGF14	HSD17B4	LITAF	NEK9	PMP2	SAMHD1	SPG11	TRPV4	
AMACR	CCDC78	DHCR24	FHL2	HSPB1	LMNA	NFU1	PMP22	SBF1	SPG20	TSEN15	
AMPD1	CCDC88C	DHTKD1	FIG4	HSPB3	LMNB1	NGF	PMPCA	SBF2	SPG21	TSEN2	
AMPD2	CCNF	DLAT	FKBP10	HSPB8	LMNB2	NHLRC1	PNKD	SCARB2	SPG7	TSEN54	
ANG	CCT5	DMD	FKBP14	HSPG2	LMOD3	NIPA1	PNKP	SCN10A	SPR	TSFM	
ANO10	CEP104	DMPK	FKRP	HTRA2	LPIN1	NKX2-1	PNPLA2	SCN11A	SPTAN1	TTBK2	
ANO3	CEP120	DMXL2	FKTN	HTT	LRP4	NOP56	PNPLA6	SCN1A	SPTBN2	TTN	
ANO5	CEP290	DNA2	FLAD1	HYLS1	LRPPRC	NOS3	PNPLA8	SCN1B	SPTLC1	TTR	
ANXA11	CEP41	DNAJB2	FLNC	IARS2	LRRK2	NOTCH3	PNPT1	SCN2A	SPTLC2	TUBA4A	
AP4B1	CFL2	DNAJB5	FLRT1	IBA57	LRSAM1	NPC1	POGLUT1	SCN4A	SQSTM1	TUBB2B	
AP4E1	CHAT	DNAJC12	FLVCR1	ICK	LTBP4	NPC2	POLG	SCN8A	STAC3	TUBB3	
AP4M1	CHKB	DNAJC13	FLVCR2	IDS	LYRM7	NPHP1	POLG2	SCN9A	STIM1	TUBB4A	
AP4S1	CHMP2B	DNAJC5	FOLR1	IFIH1	LYST	NT5C2	POLR1C	SCO1	STN1	TUFM	
AP5Z1	CHRNA1	DNAJC6	FOXRED1	IFRD1	MAG	NTRK1	POLR3A	SCO2	STUB1	TWNK	
APOA1	CHRNB1	DNM2	FRG1	IFT140	MAMLD1	NUP62	POLR3B	SCP2	STXBP1	TYMP	
APOE	CHRND	DNMT1	FTL	IFT172	MAP3K20	OCLN	POMGNT1	SCYL1	SUCLA2	TYROBP	
APOPT1	CHRNE	DOK7	FUCA1	IGHMBP2	MAPT	OPA1	POMGNT2	SDHA	SUCLG1	UBA1	
APP	CHRNG	DOLK	FUS	INF2	MARS	OPA3	POMK	SDHAF1	SUMF1	UBE3A	
APTX	CHST14	DPAGT1	G6PC	INPP5E	MARS2	OPTN	POMT1	SELENON	SURF1	UBQLN2	
AR	CHUK	DPM1	GAA	INPP5K	MATR3	ORAI1	POMT2	SEMA3A	SYNE1	UCHL1	
ARHGEF10	CIZ1	DPM2	GABRA1	IRF6	MCM3AP	P4HA1	PON2	SEPSECS	SYNE2	UNC13A	
ARL13B	CLCF1	DPM3	GABRD	ISCA2	MCOLN1	PANK2	PON3	SEPT9	SYNJ1	UQCRQ	
ARMC9	CLCN1	DRD2	GABRG2	ISCU	MECP2	PARK7	PPARGC1A	SERAC1	SYT14	UROD	
ARSA	CLCN2	DRP2	GAD1	ISPD	MECR	PAX6	PPOX	SERPINI1	SYT2	UROS	
ARSI	CLN3	DSE	GALC	ITGA7	MED25	PC	PPP2R2B	SETX	TACO1	USP8	
ASAH1	CLN5	DST	GAN	ITM2B	MEGF10	PCDH19	PPT1	SGCA	TAF1	VAC14	
ASCC1	CLN6	DSTYK	GARS	ITPR1	MFN2	PDE10A	PRDM12	SGCB	TAF15	VAMP1	
ASPA	CLN8	DUX4	GBA	JPH1	MFSD8	PDE6D	PRDM8	SGCD	TANGO2	VAPB	
ATAD3A	CLTCL1	DYNC1H1	GBA2	JPH3	MICU1	PDE8B	PREPL	SGCE	TARDBP	VCP	
ATCAY	CNBP	DYSF	GBE1	JRK	MKS1	PDGFB	PRICKLE1	SGCG	TAZ	VIPAS39	
ATL1	CNTN1	EARS2	GCDH	KARS	MLC1	PDGFRB	PRICKLE2	SGPL1	TBC1D24	VLDLR	
ATL3	CNTN2	ECHS1	GCH1	KAT6B	MME	PDHA1	PRKAG2	SH3TC2	TBCE	VMA21	
ATM	CNTNAP1	EEF2	GDAP1	KBTBD13	MORC2	PDHB	PRKCG	SIGMAR1	TBK1	VPS11	
ATN1	COASY	EFHC1	GFAP	KCNA1	MPO	PDHX	PRKN	SIL1	TBP	VPS13A	
ATP13A2	COL12A1	EGR2	GFER	KCNA2	MPV17	PDK3	PRKRA	SLC12A6	TCAP	VPS13C	
ATP1A1	COL13A1	EIF2B1	GFM1	KCNC1	MPZ	PDSS2	PRNP	SLC16A1	TCTN1	VPS33B	
ATP1A2	COL4A1	EIF2B2	GFPT1	KCND3	MR1	PDYN	PRPH	SLC17A5	TCTN2	VPS35	
ATP1A3	COL4A2	EIF2B3	GIGYF2	KCNE3	MRE11	PEX1	PRPS1	SLC18A2	TCTN3	VPS37A	
ATP2A1	COL6A1	EIF2B4	GJB1	KCNJ10	MSTN	PEX10	PRRT2	SLC18A3	TDP1	VRK1	

Table S3. Neuromuscular-related variants selected after whole-exome sequencing and software filtering

Chromosome	Position	Gene	Reference sequence	HGVSc	HGVSp	Exon	Depth (X)	dbSNP	1000Gp3_AF	SIFT	Polyphen2
19	1,043,794	*ABCA7*	NM_019112.3	c.1001G>A	p.Arg334Gln	10/47	51	rs147846250	0,000599042	T;T	B;B
4	107,253,029	*AIMP1*	NM_001142416.1	c.664C>T	p.Pro222Ser	5/7	60	rs138106524	0,000399361	D;D;D;D	B;B
1	17,318,532	*ATP13A2*	NM_022089.3	c.2097delC	p.Pro699fs	19/29	34	-	-	-	-
1	17,328,585	*ATP13A2*	NM_022089.3	c.649G>A	p.Gly217Ser	8/29	74	rs199961048	-	D;D;D;D	D;P;D
19	30,193,654	*C19orf12*	NM_001031726.3	c.424A>G	p.Lys142Glu	3/3	39	rs146170087	0,00219649	D;D;D;T	B;B
7	143,049,017	*CLCN1*	NM_000083.2	c.2926C>T	p.Arg976*	23/23	28	rs142539932	0,000599042	-	-
1	205,039,031	*CNTN2*	NM_005076.3	c.2273_2274delGGinsAT	p.Trp758Tyr	18/23	106	rs1064796187	-	-	-
13	37,581,148	*EXOSC8*	NM_181503.2	c.427G>A	p.Asp143Asn	8/11	64	rs542265577	0,000199681	T;T;T	B;B
8	75,274,121	*GDAP1*	NM_018972.2	c.487C>T	p.Gln163*	4/6	42	rs104894077	0,000199681	.;.	-
1	22,149,826	*HSPG2*	NM_001291860.1	c.13162C>T	p.Arg4388Cys	97/97	32	-	-	D	D;P
4	3,180,083	*HTT*	NM_002111.7	c.4522C>T	p.Arg1508Cys	35/67	75	rs572039089	0,000199681	T	D
9	111,693,276	*IKBKAP*	NM_003640.3	c.150+1G>A	-	2/36	35	-	-	-	-
2	8,870,884	*KIDINS220*	NM_020738.2	c.5282C>T	p.Thr1761Ile	30/30	29	-	-	D;D;D;D	B;B;B
18	6,975,995	*LAMA1*	NM_005559.3	c.6430C>G	p.Leu2144Val	45/63	30	rs117225191	0,0071885	T	D
2	11,943,118	*LPIN1*	NM_001261428.1	c.2119G>A	p.Val707Ile	16/22	12	rs146048019	-	T;T;T;T;T;T;T	B
21	47,703,705	*MCM3AP*	NM_003906.4	c.1267A>G	p.Thr423Ala	2/28	36	rs144151494	0,000798722	T;T	B
1	29,527,028	*MECR*	NM_016011.3	c.830C>T	p.Ala277Val	7/10	71	rs148978800	0,00179712	T;T	B
1	46,661,543	*POMGNT1*	NM_001243766.1	c.474G>C	p.Glu158Asp	6/23	39	-	-	T;T;T;T;T	B;B;B;B
2	179,613,347	*TTN*	NM_133379.4	c.13780G>C	p.Asp4594His	46/46	45	-	-	D	B

B: benign; T: tolerated; P: possibly damaging; D: deleterious or damaging
